# Combined spinal-epidural anesthesia for cesarean delivery in a patient with cor triloculare biventriculare

**DOI:** 10.1186/s12871-017-0411-6

**Published:** 2017-08-29

**Authors:** Yuan Han, Zhenfeng Zhang, Qingchun Sun, Ke Ding, Liu Han, Mengmeng Dong, Yifan Xu, Lei Ge

**Affiliations:** 1 Department of Anesthesiology, The Affiliated Hospital of Xuzhou Medical University, NO. 99 Huaihai Road, Quanshan District, Xuzhou city, Jiangsu Province China; 2 Jiangsu Province Key Laboratory of Anesthesiology, Xuzhou, 221002 China

**Keywords:** Cor triloculare biventriculare, Anesthesia, Heart disease, Obstetric anesthesia, Case report

## Abstract

**Background:**

Cor triloculare biventriculare accounts for approximately 0.31% of cases of congenital heart disease (CHD). Moreover, people with cor triloculare biventriculare always have shorter life spans, and cases of gestation are rare. There have been various related reports of cor triloculare biventriculare in recent years.

**Case presentation:**

We described an emergency cesarean section of a 30-year-old, 38-weeks-pregnant woman suffering from cardiac insufficiency and fetal distress. Combined spinal-epidural anesthesia was performed safely, and a male baby was smoothly delivered 10 min after the procedure without any adverse outcome on the mother or newborn. After surgery, we advised that the patient submit to an echocardiogram examination, which revealed the congenital heart disease cor triloculare biventriculare.

**Conclusions:**

Combined spinal-epidural anesthesia was administered to a patient with cor triloculare biventricularethis with good effect, ensuring the patient’s safety and meeting the need for emergency surgery.

## Background

Cor triloculare biventriculare, a rare congenital malformation of the heart in which there is a complete absence of the atrial septum, accounts for approximately 0.31% of cases of congenital heart disease (CHD) [1]. Moreover, such people always have shorter life spans, and cases of gestation are rare. General anesthesia (GA) or cautious epidural anesthesia is usually administered cases of maternal CHD via an intra-cardiac shunt. However, the effects and prognoses varied in related reports [2]. GA could provide a more stable hemodynamic state for CHD patients, but with an increased risk of pulmonary complications postoperatively. Relatively speaking, there is no risk of pulmonary complications in epidural anesthesia, but it may have a transient effect on hemodynamics. Initiation of combined spinal-epidural (CSE) anesthesia offers both rapid onset and reliable spinal anesthesia coupled with the capacity to augment or prolong the blockade through the epidural catheter. In this cor triloculare biventriculare case, we used CSE anesthesia to complete the delivery. In this study, we review our experience, addressing several key points for monitored anesthesia care as follows.

## Case presentation

A 30-year-old G4, P2 female, 160 cm tall, and weighing 68 kg, was admitted at 38 weeks of her third gestation for vaginal bleeding and hypogastralgia at her local hospital. Due to the combination of hypoxemia and abnormal electrocardiograph (ECG) findings, she was advised to transfer to our hospital; the patient did not receive prenatal obstetric care in our hospital. The admitting diagnosis was cardiac insufficiency, cyanotic CHD (dubious) and scarred uterus. The physical examination revealed blood pressure of 130/80 mmHg, a pulse of 90/min, a respiratory rate of 25/min, slightly cyanotic lips and the same on her cheeks, coarse breath sounds to auscultation in the lungs, and a regular heartbeat. A loud P2 and grade 3/6 systolic murmur was heard at the third and fourth ribs on the left edge of the sternum. The ECG showed sinus rhythm, complete right bundle branch block (CRBBB), first degree A-V block, inverted T wave, right ventricular hypertrophy, and severe cardiac clockwise rotation.

The obstetricians felt that the manifestations of cardiac insufficiency in the mother put her at high risk in a vaginal delivery. The fetus presented with abnormal heart rate variability, with no further evidence that the fetus was in a state of emergency. Considering that the mother had been in a condition of hypoxia, which would result in hypoxia in the baby, the obstetricians recommended emergency cesarean section for the benefit of both the mother and the baby.

The patient was transferred to the operating theater with a temperature of 36.8 °C, a heart rate of 92 beats/min, a respiratory rate of 23breaths/min, a blood pressure of 138/88 mmHg, and an oxygen saturation of 83-90% in air. With the administration of a fractional inspired concentration of oxygen (FiO_2_) of 0.5 to 0.6 via a Venturi mask, the patient’s oxygen saturation increased from 88 to 92%. Peripheral venous access was sited, and a 400-ml bolus of compound sodium chloride was commenced, after which a radial arterial line was placed under local anesthesia for beat-to-beat blood pressure monitoring.

Combined spinal-epidural anesthesia (single-shot spinal anesthesia) was administered in this case. The puncture point of spinal anesthesia was selected in the L2-3 interspace, and the procedure was performed using a needle-through-needle technique in the left lateral decubitus position. After cerebrospinal fluid (CSF) could be slowly aspirated through the needle, doses of 1.4 ml of 0.5% hyperbaric bupivacaine made up with 10% glucose for a hyperbaric solution that made it easy to adjust and control the anesthetic plane were injected into the subarachnoid space slowly towards the head, and an epidural catheter was inserted into the epidural space. The patient was then placed in the supine position with a left lateral tilt of at least 30 degrees; [3] the patient’s abdomen could be raised softly if necessary to relieve vena caval compression. Within 20 min later of the anesthetic injection, we tested the block height every 1-2 min and adjusted the head-up or head-down tilt position to strictly control the blockade under the level of T6-8. Twenty minutes later, the level of spinal block was static at T8. One minute later, the patient was returned to the supine position, with a slight decrease arterial pressure to 122/82 mmHg and a heart rate of 97 beats/min. As small doses of metaraminol could resist the vasodilatation caused by anesthesia to maintain the afterload, 100 μg of metaraminol was administered, after which the patient’s arterial pressure increased to 130/84 mmHg, with a heart rate of 91 beats/min.

During the entire procedure, the key for safely administering anesthesia on such a patient was to keep her blood pressure stable and to carefully address the side effects of the changes in the hemodynamics [4]. Because the spinal anesthesia took action faster and due to the rapid sympathectomy in the intra-vertebral anesthesia, great care should be taken of the changes in arterial pressure and heart rate after puncture and injection.

A male baby was smoothly delivered 10 min after the surgery began; the baby had Apgar scores of 8 at 1 min and 9 at 5 min. In addition to the caution that must be paid due to the change in the patient’s venous return at the time of the delivery of the baby, some factors need to be given attention to avoid the occurrence of great amounts of intra-cardiac shunt changes and even heart failure. First, because the oppression of the inferior vena cava was relieved after childbirth, the lower limb venous blood flow increased rapidly, and the venous return increased in a short amount of time as a result. Abdominal sandbags were adopted to add pressure to reduce the returned blood volume. Further, the maternal “autologous blood transfusion effect” had to be considered: after the fetus was delivered, the myometrium contracted, and intrauterine blood flowed back to the blood vessels rapidly, thereby increasing the cardiac preload in a short amount of time. If the initial transfusion was too great, it could lead to heart failure. According to the patient’s fluctuations in blood pressure, a bolus of ephedrine 3 mg or metaraminol 100 μg was used to restore the blood pressure to 130 ~ 140/82 ~ 87 mmHg, with a heart rate of 90 ~ 98 beats/min and an SpO_2_ of 89 ~ 93% during the surgery. The goal was to balance the preload and afterload and to try to maintain the intra-cardiac shunt in the preoperative level of stability.

Uterine atony was noted by the surgeon after delivery of the placenta. Oxytocin is usually administered to increase the contractions so as to reduce bleeding; however, a rapid infusion of oxytocin could increase the patient’s heart rate, reduce the cardiac output and coronary blood flow, and break the balance of the intra-cardiac shunt, which could lead to cardiac insufficiency. Therefore, the application of oxytocin must begin with small doses, and the dosage should be adjusted over time. Thus, oxytocin (10 mIU/min) was intravenously infused, and the infusion was adjusted in accordance with the patient’s changes in heart rate and blood pressure. Continuous post-cesarean analgesia was provided through an epidural catheter. The patient was transferred to the intensive care unit (ICU) and was then sent to the ward in stable condition the following day.

**Fig. 1:**
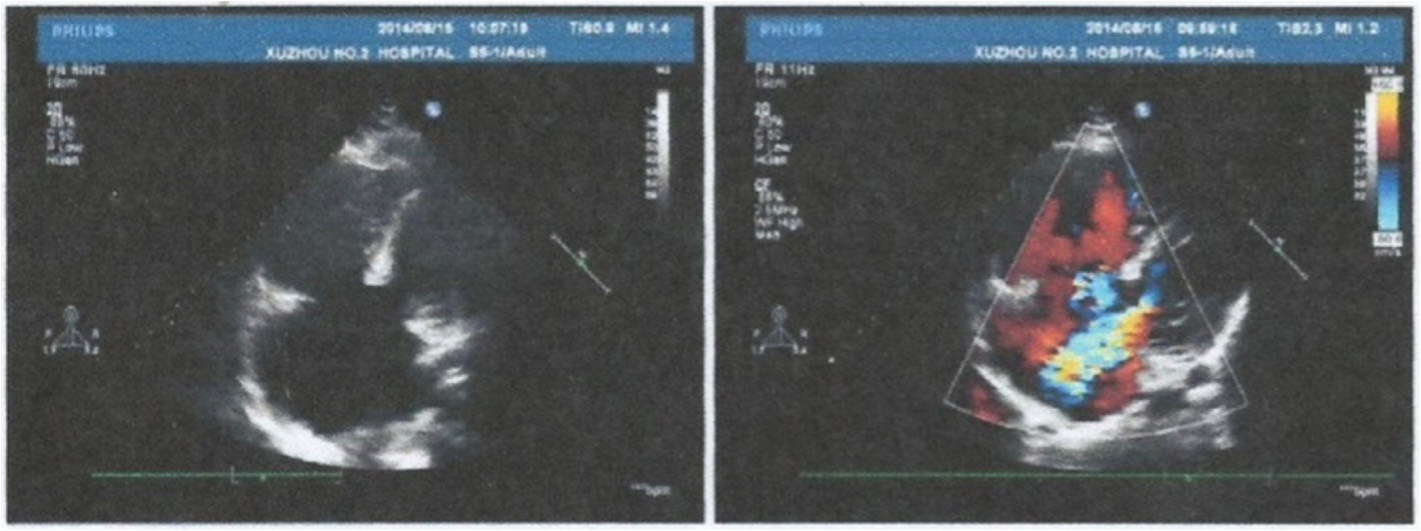
Echocardiogram of the patient’s heart, showing a single atrium. The following parameters were described: pulmonary artery, 27 mm; right ventricular diameter, 39 mm; left ventricular end diastolic diameter (LVEDD), 43 mm; left ventricular ejection fraction (LVEF), 51%; E/A, 1.01/0.69; pulmonary pressure, 16 mmHg. The echocardiogram showed evidence of congenital heart disease (CHD), including no visible nub of the interatrial septum on any section, a single atrium (SA), pulmonary artery enlargement, right ventricular hypertrophy, mitral and tricuspid valves located at the same level, poor valve development, mitral valve moderate regurgitation, tricuspid moderate regurgitation, and disappearance of the endocardial cushion decussation

We advised the patient to have an echocardiogram examination, which revealed CHD, a single atrium, and defects of the endocardial cushions (Fig. 1). Thus, we made a complementary diagnosis of CHD: single atrium. The patient was in good condition at the 6-month follow-up.

## Discussion and conclusions

Pregnancies combined with cor triloculare biventriculare are extremely rare. In this case, the patient was a multipara who suffered from cardiac insufficiency; because of fetal distress, an emergency cesarean section was performed. Before anesthesia, we (the anesthetists) did not have access to the patient’s echocardiogram results. Considering the young age of the patient and her oxygen saturation of 83-90% in air, lack of history of basic lung diseases, mild cyanosis, the detection of a loud P2 and grade 3/6 systolic murmur and the results of the ECG, we believed that she might have cyanotic CHD. As a result, we chose a cautious combined spinal-epidural anesthesia and took a series of approaches to balance the pressure of the systemic and pulmonary circulation using beat-to-beat blood pressure monitoring. Fortunately, our patient and her baby had a good outcome.

### Cor triloculare biventriculare

Cor triloculare biventriculare is a rare congenital malformation of the heart in which there is a complete absence of the atrial septum [5]. The malformation can be diagnosed by echocardiography, and the most diagnostic imaging features include disappearance of decussation – constituted by the interatrial septum, the inter-ventricular septum, mitral valve closure and tricuspid valve closure in the normal heart – and the fact that the structure of the cor triloculare biventriculare patient’s heart has an “inverted T shape”. Without the barrier of the interatrial septum, a hugely increased left-to-right shunt at the atrial level results in an obvious loading of the right ventricular volume. Pulmonary arterial hypertension (PAH) can develop early in the patient’s life, and the left-to-right shunt tends to become bidirectional or a right-to-left shunt [6]. Declining oxygen content in the systemic circulation can also develop into Eisenmenger syndrome, thereby increasing the severity of hypoxemia. Pregnancy, once Eisenmenger syndrome develops, could cause severe heart failure, pulmonary embolism, pulmonary hypertension crisis and even death [7].

### Choice of anesthesia: General versus intra-vertebral anesthesia

This delivery was an emergency cesarean section; the patient did not receive prenatal obstetric care, and she had no clear diagnosis. Because the obstetricians believed that the patient was suffering from progressive dyspnea and might progress to acute cardiac insufficiency, it was necessary to operate to reduce her cardiac load and to reduce the probability of death. Thus, our anesthetic team faced a challenge of choosing the appropriate anesthesia to ensure the patient’s safety.

In the past, anesthetists generally chose GA for anesthetic management of patients with CHD because the rapid sympathectomy and vasodilatory effects in intra-vertebral anesthesia could result in more hemodynamic instability and could worsen the right-to-left shunt. However, with the development of clinical work, anesthetists found that GA could pose some disadvantages; for instance, propofol (an anesthetic induction drug) and inhalation anesthetics can depress myocardial contractility and reduce peripheral vascular resistance, which exacerbate the right-to-left shunt. Second, increasing intrathoracic pressure by intermittent positive pressure ventilation could reduce venous return, thereby decreasing the cardiac output [7–9]. In addition, GA can also greatly increase the risk of aspiration in pregnant women. Therefore, at present, it is considered that GA should be cautiously used in pregnant women with CHD. Recent studies have shown that careful and meticulous intra-vertebral anesthesia had good outcomes in such patients, and there have been several reports of epidural anesthesia used successfully in China.

Based on the above, although anecdotal case reports of anesthetic regimens for pregnant women with specific types of CHD are available, no agreement exists on the optimal anesthetic management technique for maternal and neonatal outcomes. In this case, the patient was mildly cyanotic, and her SpO_2_ was 89 ~ 93% after oxygen. The value of the metabolic equivalent (MET) was approximately 6; overall, her condition was not severe. Therefore, we eventually performed intra-vertebral anesthesia. The key to safely perform anesthesia in such a patient is to keep the patient’s blood pressure stable and to address the side effects of hemodynamics carefully. Some key points regarding processing in monitored anesthesia care were mentioned in the case presentation section.

Spinal anesthesia is a simple and reliable technique that allowed visual confirmation of correct needle placement (by visualization of CSF) and provided rapid onset of dense neuroblockade that is typically more profound than that provided with an epidural technique, resulting in a reduced need for supplemental intravenous analgesics or conversion to general anesthesia. Only a small amount of local anesthetic was needed to establish a functional spinal blockade; therefore, spinal anesthesia is associated with negligible maternal risk for systemic local anesthetic toxicity and with minimal drug transfer to the fetus compared with epidural anesthesia. Although the spinal cord ends at L1 in most adults, it extends to the L2-L3 interspace in a small minority, so it constitutes a slight risk to select the L2-3 interspace in this case. At first, we hoped to achieve the anesthetic effect on a single spinal anesthesia, but such an approach might cause a failed block; therefore, we perform CSE anesthesia to ensure adequate analgesia. Initiation of CSE anesthesia offers both rapid onset and reliable spinal anesthesia coupled with the capacity to augment or prolong the blockade through the epidural catheter. A catheter-based technique also allows titration of the level, density, and duration of anesthesia. Indeed, a single spinal anesthesia met the requirement, and no supplementation was added via the epidural catheter. Continuous post-cesarean analgesia was provided through an epidural catheter.

The most appropriate anesthetic technique for cesarean delivery depends on maternal, fetal, and obstetric factors. In this case, the patient had not been diagnosed with Eisenmenger syndrome; the pulmonary pressure was 16 mmHg. The author thought that the choice of anesthesia in these patients was best determined by an assessment of whether the patient had Eisenmenger syndrome. Anesthetic methods and the management of patients with Eisenmenger syndrome are challenging. If the patient had been diagnosed of Eisenmenger syndrome, we are in agreement with other authors that general anesthesia is preferable for these patients [10]. A careful ‘cardiac’ GA approach might be chosen for the reason that most would consider neuraxial anesthetic a ‘risky’ option, given the risk for significant hemodynamic disturbance or a failed block intraoperatively requiring GA. In the absence of pulmonary hypertension, cautious epidural anesthesia could be considered. In conclusion, the mother and baby required optimal assessment and management. Luckily, the patient’s condition was mild, and we chose combined spinal-epidural anesthesia and were met with eventual success.

### Case review

The combined spinal-epidural anesthesia (single-shot spinal anesthesia) administered in this case provided a good effect of anesthesia, ensured the patient’s safety, and met the need for an emergency surgery. The management experience of this patient was summarized above.

Without any assessments of heart structure and function before anesthesia, we fortunately succeeded based on the patient’s mild condition. We advise that echocardiography or transthoracic echocardiography (TTE) should be performed before anesthesia, to help predict overall cardiac risk and to guide anesthetic management in pregnant women with cardiac disease.

Anesthetic selection requires further study. The current evidence on choice of anesthetic technique for such patients is based on case reports. Thus, we report our case and share it. We believe that the choice of anesthesia in these patients is best determined by an assessment of whether the patient has Eisenmenger syndrome.

## Abbreviations

CHD: Congenital heart disease
CRBBB: Complete right bundle branch block
CSE: Combined spinal-epidural
CSF: Cerebrospinal fluid
ECG: Electrocardiograph
GA: General anesthesia
ICU: Intensive care unit
PAH: Pulmonary Arterial Hypertension
TTE: Transthoracic echocardiography
